# Clinical Manifestations and Ophthalmic Outcomes of Ocular Syphilis at a Time of Re-Emergence of the Systemic Infection

**DOI:** 10.1038/s41598-018-30559-7

**Published:** 2018-08-13

**Authors:** João M. Furtado, Tiago E. Arantes, Heloisa Nascimento, Daniel V. Vasconcelos-Santos, Natalia Nogueira, Rafael de Pinho Queiroz, Luana P. Brandão, Thaís Bastos, Ricardo Martinelli, Rodrigo C. Santana, Cristina Muccioli, Rubens Belfort, Justine R. Smith

**Affiliations:** 1 Divisão de Oftalmologia, Faculdade de Medicina de Ribeirão Preto, Universidade de São Paulo, Ribeirão Preto, Brazil; 2 Departmento de Oftalmologia, Fundação Altino Ventura, Recife, Brazil; 3 Departmento de Oftalmologia e Ciências Visuais, Escola Paulista de Medicina, Universidade Federal de São Paulo, São Paulo, Brazil; 4 Departmento de Oftalmologia, Faculdade de Medicina da Universidade Federal de Minas Gerais, Belo Horizonte, Brazil; 5 Departmento de Clínica Médica, Faculdade de Medicina de Ribeirão Preto, Universidade de São Paulo, Ribeirão Preto, Brazil; 6 Flinders University College of Medicine and Public Health, Adelaide, Australia

## Abstract

Recent reports from different world regions suggest ocular syphilis is re-emerging, in parallel with an increasing incidence of the systemic infection globally. We conducted a large observational study of 127 persons consecutively treated for ocular syphilis at public medical centers in Brazil over a 2.5-year period ending July 2015. Of 104 individuals serologically tested for human immunodeficiency virus (HIV), 34.6% were positive. Ophthalmological evaluations included measurement of Snellen visual acuity and intraocular pressure, and assessment of inflammation by slit lamp examination and dilated posterior eye examination. Involvements in 214 eyes were anterior (6.1%), intermediate (8.4%), posterior (76.2%) and pan- (8.4%) uveitis, and scleritis (0.9%). Multiple anterior and posterior eye complications were observed, including cataract in the anterior eye (incidence rate, 0.18/eye-year) and epiretinal membrane in the posterior eye (incidence rate, 0.09/eye-year); incidence rates of reduction in best-corrected visual acuity to ≤20/50 and ≤20/200 were 0.10 and 0.06/eye-year, respectively. Rates of complications and visual acuity loss did not differ significantly between HIV- positive and negative individuals. In an era of re-emergence, syphilis has ocular complications that may compromise vision, despite treatment with appropriate anti-microbial drugs.

## Introduction

Over the past 3 years, the Centers for Disease Control and Prevention (CDC) have issued a series of reports, which highlight an increasing incidence of syphilis in the US. Primary and secondary syphilis have increased to a rate of 5.3 cases per 100,000 population in 2013 from the lowest recorded rate of 2.1 per 100,000 population in 2000^1^. The community at highest risk is men who have sex with men^2^, which also has an increased risk of human immunodeficiency virus (HIV) infection^3^. The increase in acquired syphilis has been accompanied by an increase in the rate of congenital syphilis, which was recorded at 11.6 cases per 100,000 live births in 2014^4^. Guidelines provided recently by the World Health Organization indicate a high incidence of this infectious disease globally^5^.

Ocular syphilis refers to the group of inflammatory eye diseases that results from infection of the ocular tissues with *Trepomena pallidum*^6,7^. Uveitis accounts for the majority of cases of ocular syphilis: characteristic presentations include acute syphilitic posterior placoid chorioretinitis^8^ and syphilitic punctate inner retinitis^9^, but diverse forms of anterior, intermediate, posterior and pan- uveitis are described. The prognosis for recovery from ocular syphilis is generally considered to be good when antimicrobial therapy is promptly instituted, but if misdiagnosis delays appropriate treatment and/or immunosuppression for presumed non-infectious inflammation is prescribed, outcomes may be quite poor^10,11^.

Reports describing large groups of patients treated at tertiary referral inflammatory eye disease services in the 1990s and 2000s indicated ocular syphilis was a rare diagnosis, accounting for less than 2% of all cases managed at these times^12–15^. In contrast, more recent reports describing cohorts of up to 85 patients with ocular syphilis in the US^11,16,17^, Europe^18–23^ and Australasia^24–27^ suggest the condition is re-emerging, consistent with the increasing incidence of the systemic infection. However, there are no current surveys of ocular syphilis in South American nations.

We investigated clinical presentations and outcomes of ocular syphilis in Brazil. Clinical information was collected for patients who presented consecutively to four public inflammatory eye disease clinics over a 2.5-year period, beginning in January 2013. With description of the disease in 214 eyes of 127 patients, our report represents the largest series of ocular syphilis cases collated to date, and provides a comprehensive description of the spectrum of this condition and its complications during the current global re-emergence of the systemic infection.

## Results

Clinical data were collected for 127 patients who presented with inflammatory eye disease that was diagnosed as ocular syphilis on the basis of a combination of serological testing, CSF analysis and/or resolution following specific antibiotic treatment. This cohort included 96 men (75.6%) and 31 women (24.4%). Although the majority of these persons were in the fifth decade of life at presentation, ages ranged widely, from 22 to 88 years. For the 104 individuals whose HIV infection status was known, 68 (65.4%) were HIV-negative and 36 (34.6%) were HIV-positive. The large majority of HIV-positive patients were men (n = 33; p = 0.006), and HIV-positive patients also were younger at presentation than HIV-negative patients by a modest interval (mean age 47.8 years versus 41.6 years, p = 0.020). Peripheral CD4+ T lymphocyte counts were available for 18 of the 36 HIV-positive patients; the mean count ± standard deviation was 558 ± 353 cells/μl (median: 527, range: 47–1500), and 2 patients had a count below 100 cells/μl. Considering all patients, symptoms of disease began a mean of 2.8 months prior to presentation. There were no significant differences in results of luetic serological testing, presence of abnormalities in the cerebrospinal fluid (CSF), and treatment with antibiotics (i.e. aqueous penicillin G or ceftriaxone, both administered intravenously) or corticosteroid between HIV-negative and HIV-positive patients. Patient characteristics are presented in Table 1.

**Table 1:** Characteristics of patients presenting with ocular syphilis (unless otherwise stated, n = 127 total patients, 68 HIV-negative patients and 36 HIV-positive patients)^a^.

Variable	All patients	HIV-negative patients	HIV-positive patients	p-value
Age (years) at diagnosis,				
mean ± SD (median, range)	46.6 ± 13.5 (48.0, 22–88)	47.8 ± 13.2 (49.0, 22–88)	41.6 ± 11.7 (41.5, 23–66)	0.020^g^
Gender, n (%)				
Female	31 (24.4)	22 (32.4)	3 (8.3)	0.006^h^
Male	96 (75.6)	46 (67.6)	33 (91.7)	
Bilateral involvement, n (%)	87 (68.5)	46 (67.6)	25 (69.4)	0.851^h^
Duration (months) of symptoms at diagnosis, mean ± SD (median, range),				
[n = 123, 67 HIV-negative, 34 HIV-positive]	2.8 ± 6.3 (1.0, 0.0–48)	2.4 ± 3.9 (1.0, 0.1–24)	3.6 ± 9.9 (1.0, 0.1–48)	0.530^i^
Positive serum treponemal test^b^, n (%)	127 (100)	68 (100)	36 (100)	NA
Titer of serum non-treponemal test^c^,				
median (range)	1:64 (1:1–1:2048)	1:64 (1:1–1:2048)	1:128 (1:1–1:1024)	0.681^i^
≤1:128, n (%)	55 (43.3)	27 (39.7)	21 (58.3)	0.070^h^
CSF abnormality^d^, n (%)				
[n = 85, 47 HIV-negative, 28 HIV-positive]	29 (34.1)	14 (29.8)	12 (42.9)	0.250^h^
Antibiotic treatment^e^, n(%) [n = 118, 65 HIV-negative, 35 HIV-positive]				
Aqueous penicillin G IV	91 (77.1)	52 (80.0)	30 (85.7)	0.478^h^
Ceftriaxone IV	27 (22.9)	13 (20.0)	5 (14.3)	
Corticosteroid treatment^f^, n(%)				
[n = 122, 67 HIV-negative, 35 HIV-positive]	74 (60.7)	41 (61.2)	17 (48.6)	0.222^h^

Abbreviations: HIV = human immunodeficiency virus; SD = standard deviation; CSF = cerebrospinal fluid; IV = intravenous; NA = not applicable. ^a^HIV testing was either not performed or results were not available for 23 patients (18.1%). ^b^Serum treponemal tests were fluorescent treponemal antibody absorption test (FTA-Abs) or microagglutination assay for *T*. *pallidum* (MHA-TP). ^c^Non-treponemal tests were Venereal Disease Research Laboratory (VDRL) and Rapid Plasmin Reagent (RPR). ^d^CSF abnormalities: positive VDRL and/or >4 leukocytes/mm^3^ and protein >40 mg/dl. CSF analysis was not available for 42 patients (33.1%). ^e^Three subjects who were treated with penicillin G benzathine and 6 subjects who failed to complete medical treatment were excluded from this analysis. ^f^Corticosteroids included oral prednisone (n = 72), periocular triamcinolone (n = 1) and intravenous methylprednisolone (n = 1). Statistical analyses were performed by: ^g^Student’s t-test; ^h^Pearson’s chi-square test; and ^i^Mann-Whitney U test.

Ocular inflammation was bilateral in 87 patients (68.5%), giving a total of 214 involved eyes. Thirteen eyes (6.1%) had anterior uveitis; 18 eyes (8.4%) had intermediate uveitis; 163 eyes (76.1%) had posterior uveitis; 18 eyes (8.4%) had panuveitis; and 2 eyes (0.9%) had isolated scleritis. Posterior uveitis was the most common type of uveitis in HIV-negative and HIV-positive persons, but anterior and intermediate forms were more common in HIV-negative individuals (9.8% and 14.3% versus 0% and 1.6% in HIV-positive, respectively), and posterior and pan- forms were more common in HIV-positive individuals (83.6% and 14.8% versus 69.6% and 6.2% in HIV-negative, respectively) (p = 0.020). Although anterior and posterior segment signs were more commonly observed on first evaluation of HIV-positive patients in comparison to HIV-negative patients, there was no significant difference in frequency of any clinical sign between the two groups. Loss of best-corrected Snellen visual acuity was also not significantly different between HIV-negative and HIV-positive patients; overall, 65.9% of individuals presented with visual acuity of 20/50 or worse, and 38.8% presented with visual acuity of 20/200 or worse. Table 2 provides a description of the ocular disease at presentation.

**Table 2:** Clinical features and best-corrected visual acuity in eyes of patients presenting with ocular syphilis (unless otherwise stated, n = 214 total eyes, 114 HIV-negative eyes and 61 HIV-positive eyes)^a^.

Variable	All eyes	HIV-negative eyes	HIV-positive eyes	p-value
Anatomic classification of uveitis^25^^b^, n (%) [n = 212 total eyes: 112 HIV-negative eyes, 61 HIV-positive eyes]				0.020^d^
Anterior	13 (6.1)	11 (9.8)	0 (0.0)	
Intermediate	18 (8.5)	16 (14.3)	1 (1.6)	
Posterior	163 (76.9)	78 (69.6)	51 (83.6)	
Panuveitis	18 (8.5)	7 (6.2)	9 (14.8)	
Anterior segment findings, n (%)				
Anterior chamber cells	96 (44.9)	43 (37.7)	33 (54.1)	0.083^d^
Keratic precipitates	42 (19.6)	17 (14.9)	14 (23.0)	0.113^d^
Posterior synechiae	24 (11.2)	13 (11.4)	8 (13.1)	0.479^d^
Cataract	19 (8.9)	14 (12.2)	5 (8.2)	0.874^d^
Conjunctival hyperemia	8 (3.7)	5 (4.4)	3 (4.9)	0.886^d^
Keratitis	4 (1.9)	2 (1.8)	2 (3.3)	0.736^e^
Nodular scleritis	3 (1.4)	3 (2.6)	0 (0.0)	0.552^e^
Posterior segment findings^c^, n (%) [n = 212 total eyes: 114 HIV-negative eyes, 60 HIV-positive eyes]				
Vitritis	94 (44.3)	48 (42.1)	28 (46.7)	0.315^d^
Papillitis	68 (32.1)	34 (29.8)	25 (41.7)	0.451^d^
Retinitis	54 (25.5)	24 (21.1)	18 (30.0)	0.194^d^
Retinal vasculitis	58 (27.4)	30 (26.3)	11 (18.3)	0.306^d^
Choroiditis/chorioretinitis	30 (14.2)	15 (13.2)	8 (13.3)	0.920^d^
Cystoid macular edema	11 (5.2)	6 (5.3)	4 (6.7)	0.939^d^
Exudative retinal detachment	9 (4.2)	5 (4.4)	4 (6.7)	0.268^d^
Epiretinal membrane	6 (2.8)	1 (0.9)	4 (6.7)	0.077^d^
Neuroretinitis	1 (0.5)	0 (0.0)	1 (1.7)	0.345^e^
Choroidal neovascularization	2 (0.9)	2 (1.8)	0 (0.0)	0.546^e^
Rhegmatogenous retinal detachment	3 (1.4)	1 (0.9)	0 (0.0)	0.999^e^
Visual acuity, n (%)				
≤20/50	141 (65.9)	75 (65.8)	40 (65.6)	0.954^d^
≤20/200	83 (38.8)	37 (32.5)	26 (42.6)	0.230^d^

Abbreviations: HIV = human immunodeficiency virus. ^a^HIV testing was either not performed or results were not available for 39 eyes (18.2%) of 23 patients. ^b^Two eyes with isolated scleritis were excluded from the classification. ^c^Evaluation of posterior segment was not possible in 2 eyes (0.9%) of 2 patients due to media opacities. Statistical analyses were performed by: ^d^generalized estimating equation models and ^e^Fisher’s exact test.

**Figure 1:**
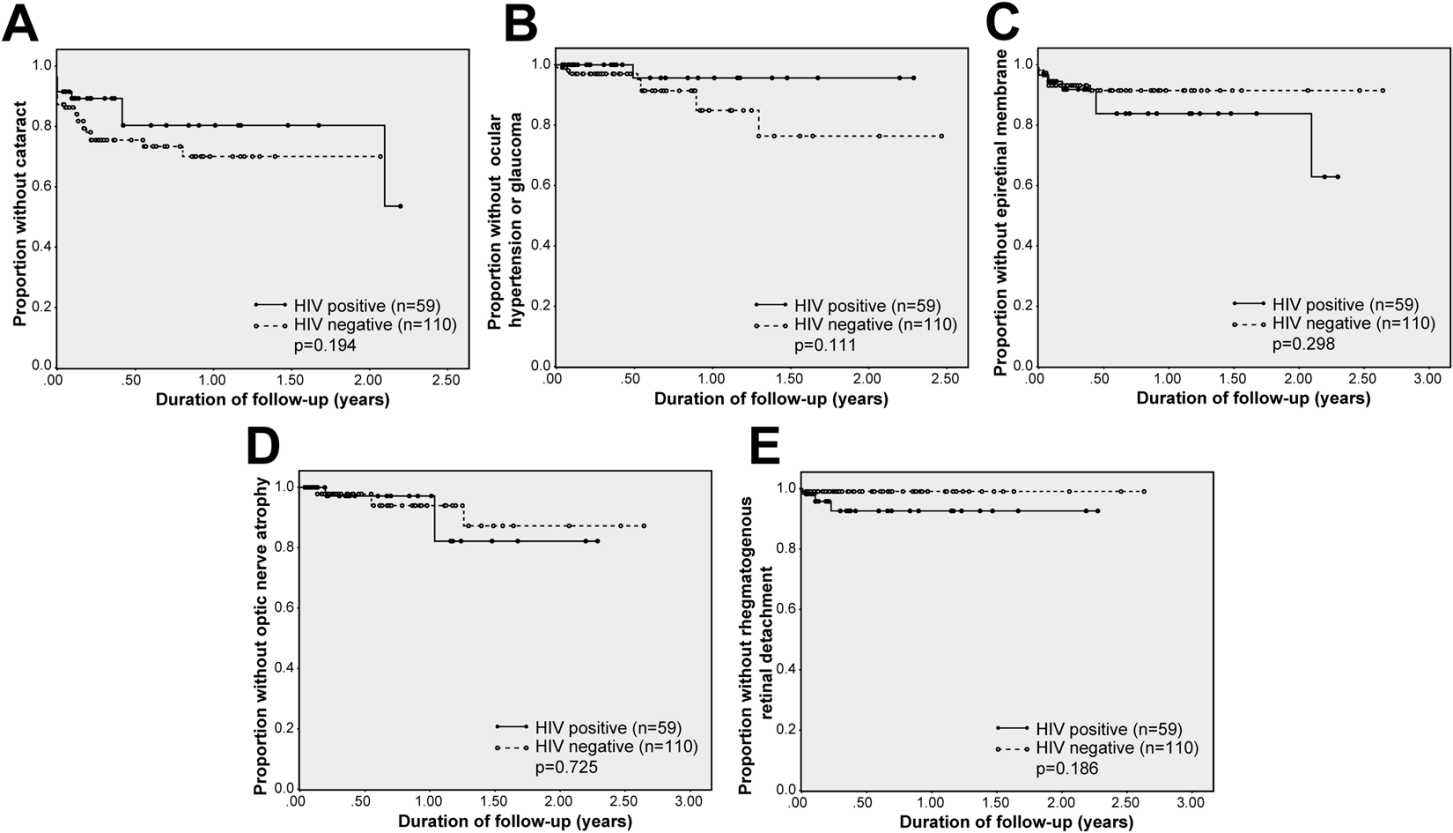
Kaplan-Meier plots showing the proportion of HIV-negative versus HIV-positive individuals with ocular syphilis who remained free of (**A**) cataract; (**B**) ocular hypertension or glaucoma; (**C**) epiretinal membrane; (**D**) optic nerve atrophy; and (**E**) rhegmatogenous retinal detachment over time, measured in years. Time 0.00 on the x-axis corresponds to the day of first ophthalmic examination.

Of the total 214 eyes, 204 eyes (95%) were evaluated in follow-up (mean follow-up interval 31.3 weeks; range 2–138 weeks). Complications reported during follow-up included cataract (incidence rate 0.18 per eye-year, 95% confidence interval [CI] 0.10–0.28 per eye-year), ocular hypertension or glaucoma (incidence rate of 0.10 per eye-year, 95% CI 0.05–0.17 per eye-year), epiretinal membrane (incidence rate of 0.09 per eye-year, 95% CI 0.04–0.16), optic nerve atrophy (incidence rate of 0.07 per eye-year, 95% CI 0.03–0.14) and rhegmatogenous retinal detachment (incidence rate of 0.05 per eye-year, 95% CI 0.02–0.11). These complications occurred in eyes of both HIV-negative and HIV-positive individuals. Incidence rates of anterior segment complications were higher in HIV-negative eyes, while incidence rates of posterior segment complications were higher in HIV-positive eyes. However, Kaplan-Meier analysis demonstrated that cumulative risks of cataract, ocular hypertension or glaucoma, epiretinal membrane, optic nerve atrophy and retinal detachment did not differ significantly between HIV-positive and HIV-negative patients. No cases of cystoid macular edema, choroidal neovascularization or occlusions of major retinal vessels were diagnosed in either patient group during follow-up. Incidence rates of ocular complications appear in Table 3, and Kaplan-Meier plots of cumulative risks are presented as Fig. 1.

**Table 3:** Incidence rates of ocular complications and best-corrected visual acuity loss for eyes of patients diagnosed with ocular syphilis^a^ (n = 204 total eyes for which there was follow-up, 110 HIV-negative eyes and 59 HIV-positive eyes).

Variable	All eyes				HIV-negative eyes				HIV-positive eyes			
	Number at risk	Number of events	Incidence rate (per eye-year)	Poisson Exact CI 95%	Number at risk	Number of events	Incidence rate (per eye-year)	Poisson Exact CI 95%	Number at risk	Number of events	Incidence rate (per eye-year)	Poisson Exact CI 95%
Cataract	185	16	0.18	0.10–0.28	96	12	0.26	0.14–0.46	54	4	0.15	0.04–0.39
Glaucoma/Ocular hypertension	202	11	0.10	0.05–0.17	109	8	0.13	0.05–0.25	59	1	0.03	0.0004–0.16
Epiretinal membrane	200	11	0.09	0.04–0.16	109	6	0.09	0.03–0.19	57	5	0.15	0.05–0.36
Optic nerve atrophy	204	9	0.07	0.03–0.14	110	5	0.07	0.02–0.16	59	3	0.09	0.02–0.26
Rhegmatogenous retinal detachment	201	6	0.05	0.02–0.11	109	1	0.01	0.0001–0.07	59	3	0.09	0.02–0.27
Cystoid macular edema	191	0	0	NA	104	0	0	NA	54	0	0	NA
Choroidal neovascularization	200	0	0	NA	108	0	0	NA	58	0	0	NA
Occlusion of major retinal vessel	202	0	0	NA	109	0	0	NA	59	0	0	NA
**Visual acuity**												
≤20/50	69	4	0.10	0.03–0.25	37	2	0.08	0.009–0.30	21	2	0.20	0.02–0.71
≤20/200	126	5	0.06	0.02–0.14	74	3	0.06	0.001–0.18	35	2	0.10	0.01–0.35

Abbreviation: NA = not applicable. ^a^The format used in this table follows the format employed by Moradi *et al*.^11^.

As presented in Table 2, best-corrected visual acuity at presentation ranged widely, but was reduced in the majority of the 214 eyes. Initial visual acuity was 20/50 or worse in 141 eyes (65.9%) and 20/200 or worse in 83 eyes (38.8%). Numbers of patients with visual acuity at or below 20/50 or 20/200 at presentation did not differ significantly between HIV-negative and HIV-positive persons. Incidence rate of vision reduction to 20/50 or worse was 0.10 per eye-year (95% CI 0.03–0.25): rates were 0.08 per eye-year (95% CI 0.009–0.30) for HIV-negative persons and 0.20 per eye-year (95% CI 0.02–0.71) for HIV-positive persons. Incidence rate of vision reduction to 20/200 or worse was 0.06 per eye-year (95% CI 0.02–0.14): rates were 0.06 per eye-year (95% CI 0.001–0.18) for HIV-negative persons and 0.10 per eye-year (95% CI 0.01–0.35) for HIV-positive persons. Incidence rates of visual acuity loss appear in Table 3. During follow-up, a 2-line minimum improvement in Snellen visual acuity was recorded in 80 of 162 eyes (45.7%), and a 2-line minimum decrease was recorded in 8 eyes (4.9%). By the 4–8 week visit, 95 of 162 eyes (58.2%) saw 20/40 or better, 67 eyes (41.4%) saw 20/50 or worse, and 27 eyes (16.7%) saw 20/200 or worse. There was no significant difference in visual acuity at the 4–8 week visit, or in change in visual acuity between individuals who were HIV-negative or HIV-positive. Best-corrected visual acuity and visual acuity change during follow-up are presented in Tables 4 and 5, respectively.

**Table 4:** Best-corrected visual acuity at presentation and during follow-up for eyes of patients diagnosed with ocular syphilis.

Visual acuity, n (%)	Time point			
	Presentation (n = 214 eyes)	2 week visit (n = 196 eyes)	4–8 week visit (n = 162 eyes)	Last visit^a^ (n = 196 eyes)
≥20/40	73 (34.1)	114 (58.2)	95 (58.6)	118 (60.2)
≤20/50	141 (65.9)	82 (41.8)	67 (41.4)	78 (39.9)
≤20/200	83 (38.8)	34 (17.3)	27 (16.7)	35 (17.9)

^a^Last visit was at 31.3 ± 31.6 weeks (mean ± standard deviation) or 19.2, 2–138 weeks (median, range).

**Table 5:** Best-corrected visual acuity and visual acuity change from presentation for eyes of patients diagnosed with ocular syphilis (n = 162 total eyes, 92 HIV-negative eyes and 46 HIV-positive eyes)^a^ at 4–8 week follow-up visit.

Visual acuity, n (%)	All eyes	HIV-negative eyes	HIV-positive eyes	p-value^b^
≤20/50	67 (41.4%)	39 (42.4%)	20 (43.5%)	0.978
≤20/200	27 (16.7%)	13 (14.1%)	11 (23.9%)	0.296
Change				0.898
No change	74 (45.7%)	45 (48.9%)	20 (43.5%)	
Increase (≥2 lines)	80 (49.4%)	43 (46.7%)	22 (47.8%)	
Decrease (≥2 lines)	8 (4.9%)	4 (4.3%)	4 (8.7%)	

Abbreviations: HIV = human immunodeficiency virus; NA = not applicable. ^a^HIV testing was either not performed or results were not available for 24 eyes (14.8%) of 14 patients. ^b^Statistical analyses were performed by generalized estimating equation models.

## Discussion

Re-emergence of syphilis since approximately 2000 is well documented by national and international surveillance bodies^1–3,5^. The change in epidemiology has been attributed to factors that include high-risk sexual practices and global travel, immunomodulatory impacts of infection with HIV and highly active anti-retroviral therapy, and changes in antibiotic sensitivity of *T*. *pallidum*^28–30^. A recent plethora of case series of ocular syphilis^11,18–21,23–27^ has suggested a re-emergence of ocular syphilis, although studies using national databases in the US and UK^22,31^ have not supported an increase in incidence. As discussed by the authors of those studies, heightened awareness and early treatment of syphilis might be relevant; however, a temporal lag might also explain this discrepancy, with both studies including data collected up to 2011.

Our investigation was conducted between January 2013 and July 2015. Two pieces of evidence suggest the incidence of ocular syphilis is increasing in Brazil. The Escola Paulista de Medicina - Hospital São Paulo has published two surveys of consecutive patients presenting to the uveitis service. In the first survey^15^, conducted between 1975 and 1979, ocular syphilis was responsible for 1.8% of presentations; in the second survey^32^, conducted from 2012 to 2013, ocular syphilis was responsible for 6.1% of presentations. Separately, review of the cases of the ocular syphilis managed at Hospital das Clínicas de Ribeirão Preto, a referral center for this study and home to the only public inflammatory eye disease clinic in the region, indicated 10 cases between 2000 and 2012 (0.77 cases/year) and 25 cases between 2013 and 2015 (8.33/year); there were no changes in the referral system and no expansion of patient services that might otherwise explain this increase.

We report the largest series of ocular syphilis yet described, including 127 patients diagnosed by standard criteria including serological testing, CSF analysis and response to anti-microbial treatment. Just over three-quarters of our patients were men. Age at presentation ranged widely, but averaged in the fifth decade. Approximately one-third of the patients were HIV-positive. These demographics are consistent with those reported by three smaller case series of patients with syphilitic uveitis: a cohort of 85 persons from The Netherlands^19^, a cohort of 66 persons from France^21^, and a cohort of 50 persons from Spain^20^. In those studies, 62–92% of patients were men; mean age varied between 41 and 49 years; and 34–47% of patients were HIV-positive. In keeping with findings of British Ocular Syphilis Study^22^, which reported on 41 persons diagnosed between 2009 and 2011, uveitis was by far the most common presentation of ocular syphilis in our cohort, affecting 212 of 214 eyes. Posterior uveitis – typically characterized by retinal involvement – was the most common uveitis subtype, accounting for 77%, while anterior, intermediate and pan- uveitis each accounted for less than 10%.

There is debate regarding the optimum management of ocular syphilis, including indications for lumbar puncture, anti-microbial treatment, and use of adjunctive corticosteroid^33^. Lumbar puncture, performed in two-thirds of our patients, demonstrated abnormalities in almost 30% of these individuals. A new report from South Africa on the use of lumbar puncture in the management of ocular syphilis^34^ indicated 46% of 68 patients diagnosed with ocular syphilis were investigated with lumbar puncture, and one-quarter of those investigated had findings consistent with neurosyphilis, including positive FTA-ABS and/or lymphocytic pleocytosis. On the basis of their results, the authors concluded, “lumbar puncture should be a routine investigation for all patients diagnosed with ocular syphilis”. Over 90% of our patients were treated with IV aqueous penicillin G or ceftriaxone, following the CDC recommendations for treatment of ocular syphilis^35^, which are identical to those for neurosyphilis. These recommendations have been widely adopted, based on recent reports^11,17–19,23,25–27^. There are no guidelines for the use of corticosteroid medications in patients with ocular syphilis, although these drugs may be given in conjunction with antibiotics to limit inflammation;^11,18,19,25–27^ approximately 60% of our patients received locally injected or systemic corticosteroid. Interestingly, in the study of 66 French patients with syphilitic uveitis, persistence of inflammation one month after commencement of treatment was associated with use of intravenous or periocular, but not oral or topical, corticosteroid^21^.

In keeping with the diverse clinical involvements, we documented a wide range of complications in the eyes with ocular syphilis, on presentation and/or during follow-up. Cataract was the most common anterior segment complication, seen initially in 9% of affected eyes and having an incidence rate of 0.18 per eye-year. Ocular hypertension or glaucoma occurred with an incidence rate of 0.10 per eye-year. Regarding posterior segment complications, cystoid macular edema was first observed only at presentation and in 5% of involved eyes, while epiretinal membrane was seen initially in 3% of affected eyes, but also appeared during follow-up with an incidence rate of 0.09 per eye-year. Although retinal vasculitis was noted in more than one-quarter of eyes with ocular syphilis, occlusions of major retinal vessels did not result. The most comprehensive published report of complications in ocular syphilis prior to our report describes 35 US patients managed at John Hopkins School of Medicine^11^. A similar range of complications was observed in these patients, albeit with different initial frequencies and incidence rates, probably reflecting different cohort demographics (i.e. two-thirds African-American and 54% HIV-positive), in addition to the shorter interval between onset of uveitis and presentation.

We recorded reduced visual acuities at presentation for a majority of eyes with ocular syphilis: 39% of eyes registered visual acuities of 20/200 or worse, and another 27% of eyes had visual acuities between 20/50 and 20/100. Although approximately one-half of eyes had a 2-line or more improvement of best-corrected Snellen visual acuity during follow-up, 17% of eyes remained 20/200 or worse and another 25% of eyes were between 20/50 and 20/100 at the 4–8 week evaluation. The incidence rate for vision loss to 20/50 or worse was 0.10 per eye-year and for vision loss to 20/200 or worse was 0.06 per eye-year. Differences in reporting of visual acuity make it difficult to compare our findings with those of studies from The Netherlands, France and Spain^19–21^. However, each of those studies reported improvement in visual acuity after treatment in the majority, but with some patients having persistent vision loss. That approximately 40% of our patients were left with reduced visual acuity might reflect the mean 3-month time interval for diagnosis of ocular syphilis. In a cohort of 20 HIV-positive Japanese patients^27^, presence of ocular symptoms for more than one month prior to diagnosis was associated with poor final visual acuity.

Given the strong link between HIV infection and ocular syphilis, it is relevant to compare presentation and course in HIV-negative versus HIV-positive individuals. There were no differences in drug treatments and time to treatment in our HIV-positive and HIV-negative patients. We observed a younger age and higher number of males in the HIV-positive group. We also observed more posterior and pan- uveitis, and less anterior and intermediate uveitis, in HIV-positive persons in comparison to HIV-negative persons. This distribution of uveitis by HIV infection status is supported by independent analyses of the literature^36,37^. Incidence rates of complications reflected the anatomic location of the uveitis – lower for anterior segment complications and higher for posterior segment complications in HIV-positive eyes – and incidence rates for visual acuity loss were higher in HIV-positive persons. However, rates of vision loss, as well as changes in visual acuity in follow-up, were equivalent to those seen in HIV-negative individuals. In the cohorts of 50 Spanish patients^20^ and 41 UK patients^22^, visual acuity outcomes between HIV-negative and HIV-positive patients were also similar.

We have reported a large observational case series of ocular syphilis, including patients diagnosed during a 2.5-year period that ended July 2015, in four tertiary referral inflammatory eye disease clinics in Brazil. Strengths of our research include substantial sample size, recent and relatively short time interval of enrollment, and the process of standardized data collection on consecutive cases. Limitations of the work reflect its retrospective nature, including in particular, availability of some information, such HIV infection status and results of CSF analysis, for a limited subset of subjects. Although our clinics receive tertiary referrals, they are based at public hospitals; since at least 75% of the Brazilian population uses the public health system^38^, our data are likely to be reflective of ocular syphilis as it occurs in the general population. Our results indicate that today ocular syphilis most commonly manifests as posterior uveitis, and frequently results in complications that may compromise vision, despite treatment with appropriate antibiotics. Medical practitioners should consider the possibility of, and investigate for, syphilis in all cases of uveitis.

## Methods

Uveitis-specialized ophthalmologists collected clinical data from consecutive cases of ocular syphilis diagnosed between January 2013 and July 2015 inclusive, at tertiary referral inflammatory eye disease clinics based at four public medical institutions in Brazil: Hospital das Clínicas de Ribeirão Preto, Fundação Altino Ventura, Escola Paulista de Medicina - Hospital São Paulo and Hospital São Geraldo/Hospital das Clínicas da UFMG. Data were recorded in a standardized manner, using a data collection sheet that was approved by the human ethics committee at each institution (Comitê de Etica em Pesquisa do Hospital das Clínicas de Ribeirão Preto, Comitê de Ética em Pesquisa da Fundação Altino Ventura, Comitê de Etica em Pesquisa da UFMG e Comitê de Etica em Pesquisa da UNIFESP/Hospital São Paulo), with the identity of each subject removed for collation and analysis of the clinical data. All experiments were performed in accordance with relevant guidelines and regulations. Since medical records were retrospectively analyzed, informed consent from subjects was not obtained.

The diagnosis of ocular syphilis was made on findings of ocular inflammation by ophthalmological examination and confirmation of systemic infection with *T*. *pallidum*. Systemic infection was indicated by a reactive treponemal serological test (i.e. fluorescent treponemal antibody absorption test [FTA-ABS] or microhemagglutination assay for *T*. *pallidum* [MHA-TP]), in addition to: (1) a reactive non-treponemal serological test (i.e. venereal disease research laboratory test [VDRL] or rapid plasmin reagent test [RPR]); (2) an abnormal CSF (i.e. reactive VDRL and/or greater than 4 leukocytes/mm^3^ and protein concentration less than 40 mg/dl); and/or (3) consistent clinical signs that resolved following intravenous treatment with aqueous penicillin G or ceftriaxone.

Subject age, gender and presence of HIV infection were recorded. Details of the clinical presentation were collected, including duration of ophthalmic symptoms at diagnosis, laterality of uveitis, and ophthalmological findings at diagnosis (i.e. best-corrected Snellen visual acuity, intraocular pressure, and assessment of ocular inflammation and complications by slit lamp examination and dilated fundoscopy). Uveitis was classified according to the Anatomic Classification of Uveitis, as described in the Standardization of Uveitis Nomenclature (SUN) for Reporting Clinical Data^39^. Details of medical management, and visual acuity and ocular complications during follow-up were recorded. Cataract was defined as any degree of lens opacification by slit lamp examination after pupillary dilation. Ocular hypertension was recorded when intraocular pressure measured over 21 mmHg by Goldmann applanation tonometry, and glaucoma was diagnosed if the raised intraocular pressure was associated with optic nerve fiber layer thinning and/or typical visual field defects. A clinical diagnosis of epiretinal membrane was verified by optical coherence tomography (OCT), and presence of cystoid macular edema, choroidal neovascularization and retinal vascular occlusion was confirmed by OCT and/or fluorescein angiography.

Statistical analyses were performed using SPSS 16.0 for Windows (SPSS, Inc, Chicago, IL). Continuous variables were expressed as mean plus/minus standard deviation (SD), or median and range. Categorical variables were expressed as absolute and relative frequencies. Pearson’s chi-square test, Student’s t-test and Mann-Whitney U test were used to assess differences between clinical variables for HIV-positive and HIV-negative individuals. For subjects with bilateral involvement, both eyes were included in analyses, and generalized estimating equation (GEE) models were implemented to account for inter-eye correlations. Fisher’s exact test was used when sample size was too small for a GEE model. Incidence rate for each ocular complication was calculated as number of events divided by sum of eye-years of at-risk eyes, and Poisson 95% confidence intervals (CIs) were calculated for incidence rates. Cumulative risk of an ocular complication was estimated using the Kaplan-Meier method and compared using the log-rank test. A p-value less than 0.05 defined a statistically significant difference. All data generated or analysed during this study are included in this published article.

## References

[1] Patton ME (2014). Primary and secondary syphilis–United States, 2005–2013. MMWR Morb Mortal Wkly Rep..

[2] Centers for Disease Control and Prevention. Sexually Transmitted Disease Surveillance 2015. Atlanta: U.S. Department of Health and Human Services; Accessed 17/11/2016 (2016).

[3] Centers for Disease Control and Prevention. HIV Infection Risk, Prevention, and Testing Behaviors among Men Who Have Sex With Men - National HIV Behavioral Surveillance, 20 U.S. Cities, 2014. HIV Surveillance Special Report 15. Accessed 11/17/2016 (2016).

[4] Bowen V, Su J, Torrone E, Kidd S, Weinstock H (2015). Increase in incidence of congenital syphilis - United States, 2012–2014. MMWR Morb Mortal Wkly Rep..

[5] World Health Organization. WHO Guidelines for the Treatment of Treponema pallidum (Syphilis). Geneva (2016). 27631046

[6] Davis JL (2014). Ocular syphilis. Curr Opin Ophthalmol..

[7] Margo CE, Hamed LM (1992). Ocular syphilis. Surv Ophthalmol..

[8] Eandi CM (2012). Acute syphilitic posterior placoid chorioretinitis: report of a case series and comprehensive review of the literature. Retina..

[9] Wickremasinghe S (2009). Syphilitic punctate inner retinitis in immunocompetent gay men. Ophthalmology..

[10] Afonso VC (2015). Visual loss resulting from immunosuppressive therapy in patients with syphilitic uveitis. Arq Bras Oftalmol..

[11] Moradi A (2015). Clinical features and incidence rates of ocular complications in patients with ocular syphilis. Am J Ophthalmol..

[12] Rodriguez A (1996). Referral patterns of uveitis in a tertiary eye care center. Arch Ophthalmol..

[13] Jakob E (2009). Uveitis subtypes in a german interdisciplinary uveitis center–analysis of 1916 patients. J Rheumatol..

[14] Rathinam SR, Namperumalsamy P (2007). Global variation and pattern changes in epidemiology of uveitis. Indian J Ophthalmol.

[15] Abreu MT, Hirata PS, Belfort R, Domingues Neto S (1980). Uveites em Sao Paulo. Estudo epidemiologico, clinico e terapeutico. Arq Bras Oftalmol..

[16] Oliver SE (2016). Ocular Syphilis - Eight Jurisdictions, United States, 2014–2015. MMWR Morb Mortal Wkly Rep..

[17] Lee SY, Cheng V, Rodger D, Rao N (2015). Clinical and laboratory characteristics of ocular syphilis: a new face in the era of HIV co-infection. J Ophthalmic Inflamm Infect..

[18] Balaskas K, Sergentanis TN, Giulieri S, Guex-Crosier Y (2011). Analysis of significant factors influencing visual acuity in ocular syphilis. Br J Ophthalmol..

[19] Bollemeijer JG (2016). Clinical manifestations and outcome of syphilitic uveitis. Invest Ophthalmol Vis Sci..

[20] Fonollosa A (2016). Clinical manifestations and outcomes of syphilis-associated uveitis in Northern Spain. Ocul Immunol Inflamm..

[21] Hoogewoud F (2017). Prognostic Factors in Syphilitic Uveitis. Ophthalmology..

[22] Mathew RG, Goh BT, Westcott MC (2014). British Ocular Syphilis Study (BOSS): 2-year national surveillance study of intraocular inflammation secondary to ocular syphilis. Invest Ophthalmol Vis Sci..

[23] Sahin O, Ziaei A (2016). Clinical and laboratory characteristics of ocular syphilis, co-infection, and therapy response. Clin Ophthalmol..

[24] Kim Y, Yu SY, Kwak HW (2016). Non-human immunodeficiency virus-related ocular syphilis in a Korean population: clinical manifestations and treatment outcomes. Korean J Ophthalmol..

[25] Northey LC, Skalicky SE, Gurbaxani A, McCluskey PJ (2015). Syphilitic uveitis and optic neuritis in Sydney, Australia. Br J Ophthalmol..

[26] Yang P, Zhang N, Li F, Chen Y, Kijlstra A (2012). Ocular manifestations of syphilitic uveitis in Chinese patients. Retina..

[27] Tsuboi M (2016). Prognosis of ocular syphilis in patients infected with HIV in the antiretroviral therapy era. Sex Transm Infect..

[28] Arora N (2016). Origin of modern syphilis and emergence of a pandemic Treponema pallidum cluster. Nat Microbiol..

[29] Rekart ML (2017). A double-edged sword: does highly active antiretroviral therapy contribute to syphilis incidence by impairing immunity to Treponema pallidum?. Sex Transm Infect..

[30] Stamm LV (2016). Syphilis: Re-emergence of an old foe. Microb Cell..

[31] Albini T (2017). Trends in hospitalization and incidence rate for syphilitic uveitis in the United States from 1998 to 2009. Am J Ophthalmol..

[32] Gonzalez Fernandez D, Nascimento H, Nascimento C, Muccioli C, Belfort R (2017). Uveitis in Sao Paulo, Brazil: 1053 new patients in 15 months. Ocul Immunol Inflamm..

[33] Tuddenham S, Ghanem KG (2016). Ocular syphilis: opportunities to address important unanswered questions. Sex Transm Infect..

[34] Reekie, I. & Reddy, Y. Use of lumbar punctures in the management of ocular syphilis. Semin Ophthalmol. (in press). 10.1080/08820538.2016.1228986 27860537

[35] Centers for Disease Control and Prevention. Syphilis - 2015 STD Treatment Guidelines. Atlanta: U.S. Department of Health and Human Services; Accessed 12/7/2017 (2016).

[36] Amaratunge BC, Camuglia JE, Hall AJ (2010). Syphilitic uveitis: a review of clinical manifestations and treatment outcomes of syphilitic uveitis in human immunodeficiency virus-positive and negative patients. Clin Exp Ophthalmol..

[37] Tucker JD (2011). Ocular syphilis among HIV-infected patients: a systematic analysis of the literature. Sex Transm Infect..

[38] Paim J, Travassos C, Almeida C, Bahia L, Macinko J (2011). The Brazilian health system: history, advances, and challenges. Lancet..

[39] Jabs DA, Nussenblatt RB, Rosenbaum JT (2005). & Standardization of Uveitis Nomenclature Working. Standardization of uveitis nomenclature for reporting clinical data. Results of the First International Workshop. Am J Ophthalmol.

