# Author Correction: Clinical Manifestations and Ophthalmic Outcomes of Ocular Syphilis at a Time of Re-Emergence of the Systemic Infection

**DOI:** 10.1038/s41598-018-34170-8

**Published:** 2018-10-23

**Authors:** João M. Furtado, Tiago E. Arantes, Heloisa Nascimento, Daniel V. Vasconcelos-Santos, Natalia Nogueira, Rafael de Pinho Queiroz, Luana P. Brandão, Thaís Bastos, Ricardo Martinelli, Rodrigo C. Santana, Cristina Muccioli, Rubens Belfort, Justine R. Smith

**Affiliations:** 1 Divisão de Oftalmologia, Faculdade de Medicina de Ribeirão Preto, Universidade de São Paulo, Ribeirão Preto, Brazil; 2 Departmento de Oftalmologia, Fundação Altino Ventura, Recife, Brazil; 3 Departmento de Oftalmologia e Ciências Visuais, Escola Paulista de Medicina, Universidade Federal de São Paulo, São Paulo, Brazil; 4 Departmento de Oftalmologia, Faculdade de Medicina da Universidade Federal de Minas Gerais, Belo Horizonte, Brazil; 5 Departmento de Clínica Médica, Faculdade de Medicina de Ribeirão Preto, Universidade de São Paulo, Ribeirão Preto, Brazil; 6 Flinders University College of Medicine and Public Health, Adelaide, Australia

Correction to: *Scientific Reports* 10.1038/s41598-018-30559-7, published online 13 August 2018

This Article contains an error in the Methods section.

“(2) an abnormal CSF (i.e. reactive VDRL and/or greater than 4 leukocytes/mm^3^ and protein concentration less than 40 mg/dl);”

should read:

“(2) an abnormal CSF (i.e reactive VDRL and/or greater than 4 leukocytes/mm^3^ and protein concentration greater than 40 mg/dl);”

